# Correlation of Fine Needle Aspiration Cytology Findings with Thyroid Function Test in Cases of Lymphocytic Thyroiditis

**DOI:** 10.1155/2014/430510

**Published:** 2014-04-06

**Authors:** Neelam Sood, Jitendra Singh Nigam

**Affiliations:** ^1^Department of Pathology, D.D.U. Hospital, Harinagar, New Delhi, India; ^2^Department of Pathology, Saraswathi Institute of Medical Sciences, Anwarpur, Pilkhuwa, Hapur, Uttar Pradesh 245304, India

## Abstract

*Background*. Chronic lymphocytic thyroiditis is the second most common thyroid lesion diagnosed on FNAC after goiter. FNAC is reliable tool in the diagnosis of thyroid lesion. *Objective*. To correlate FNAC cytologic findings with TFT in the lymphocytic thyroiditis. *Methods*. 175 patients with thyroid swellings were referred for FNAC as well as TFT during 2011–2013. Fine needle aspiration cytology was performed using non-aspiration or aspiration techniques and TFT performed on Beckman culter access 2. *Results*. Lymphoid infiltrate was seen in 55 cases. The commonest age group of lymphocytic thyroiditis was 21–30 years with male : female ratio being 1 : 10. Anti-TPO and TSH were elevated in 96.16% (25/26) of cases with grade 3 lymphoid infiltrate, 94.12% (16/17) of cases with grade 2, and 91.67% (11/12) of cases with 1 grade. Increased anti-TPO with raised TSH without any lymphoid infiltrate was seen in 5 cases and 5 cases showed only raised TSH without raised anti-TPO and without any lymphoid infiltrate. We observed that grade 3 lymphocytic infiltration has correlation with anti-TPO and TSH together or TSH alone but not with anti-TPO alone. We also observed that anti-TPO and TSH together are significant even if no lymphocytic infiltration is present. *Conclusion*. Grade 3 lymphocytic infiltration has statistical correlation with anti-TPO and TSH together or TSH alone but not with anti-TPO alone. Anti TPO was adjunct to TSH in grade 3. The presence of Hurthle cell change, giant cells, and granulomas has no statistical correlation with lymphocytic thyroiditis.

## 1. Introduction

Hashimoto's thyroiditis, a synonym of chronic lymphocytic thyroiditis or autoimmune thyroiditis, is the second most common thyroid lesion diagnosed on FNAC after goiter [1, 2]. Hurthle cell change and an increased number of mature and transformed lymphocytes impinging on follicular cells is the characteristic of Hashimoto's thyroiditis [2]. Hashimoto's thyroiditis is more common in women and has prevalence rate of 1–4% and incidence of 30–60/100000 population per year [1]. In 1960 Schade et al. analysed the relationship between thyroid autoimmunity and the presence of lymphocytes in the thyroid gland in patients with Graves' disease, toxic adenoma, and nontoxic nodular goitre and found that in all these conditions circulating antibody to thyroglobulin was significantly associated with lymphocytic infiltration [3]. Cytological grading on FNAC smears using predefined sets of criteria was for the first time done by Bhatia et al. and tried to correlate the lymphoid density with clinical, radiological, and biochemical parameters [2]. In the present study, cytomorphologic features of thyroid FNAC were graded according to lymphocyte infiltrate as per the criteria mentioned in Table 1 [2] and correlate with TFT.

## 2. Material and Methods

175 patients with thyroid swellings were referred to department of pathology for FNAC as well as TFT during 2011–2013. Out of 175 patients, 159 were females and 16 were males. Colloid goiter was the commonest diagnosis (59/175) followed by lymphocytic thyroiditis (55/175). FNAC was performed using nonaspiration or aspiration techniques by 23 G needle with 10 mL syringe. Air-dried smears were stained with Giemsa stain and wet ethanol fixed smears were stained with hematoxylin and eosin. TFT was performed on Beckman coulter access 2. Cytomorphologic features were reviewed and graded according to lymphocyte infiltrate and other parameters, for example, presence of granuloma, Hurthle cells, degree of anisonucleosis, giant cells, and so forth. These were statistically analysed for correlation with anti-thyroid peroxidase (anti-TPO) antibodies and hypersensitive TSH.

## 3. Results

**Figure 1:**
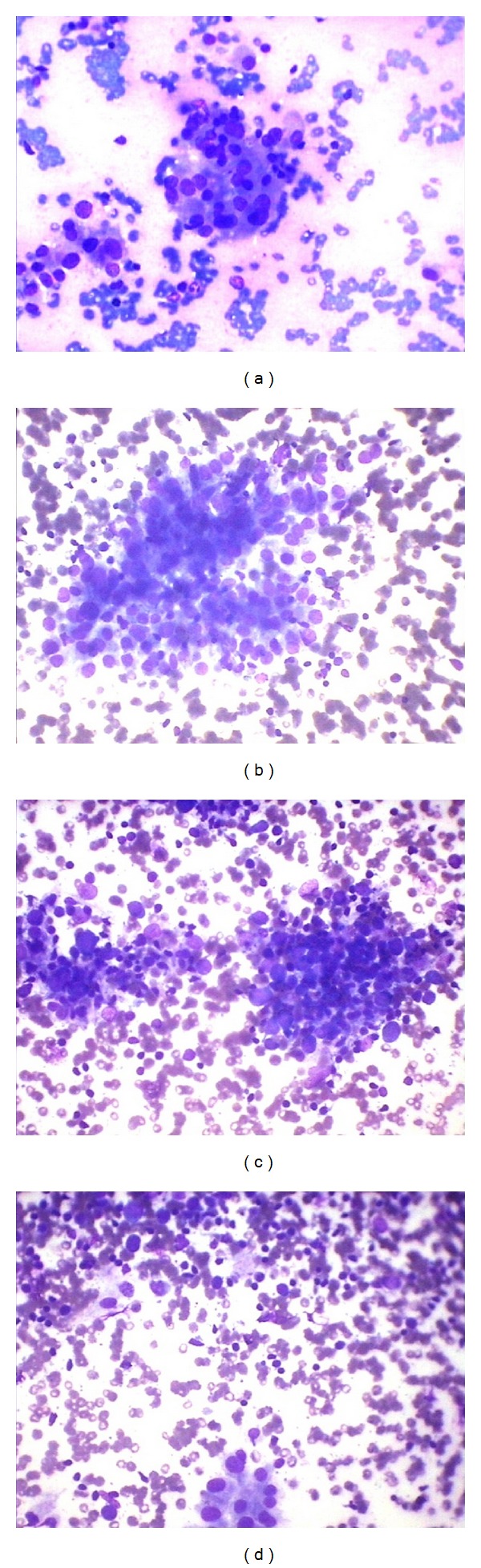
(a) Grade I: cluster of follicular epithelial cells infiltrated by few lymphoid cells (Giemsa ×400). (b) Grade II: cluster of follicular epithelial cells infiltrated by significant lymphoid cells (Giemsa ×400). (c) Grade III: total destruction of follicle with dense infiltration by lymphoid cells (Giemsa ×400). (d) Grade III: lymphoid infiltrate with residual follicular epithelial cells.

**Figure 2:**
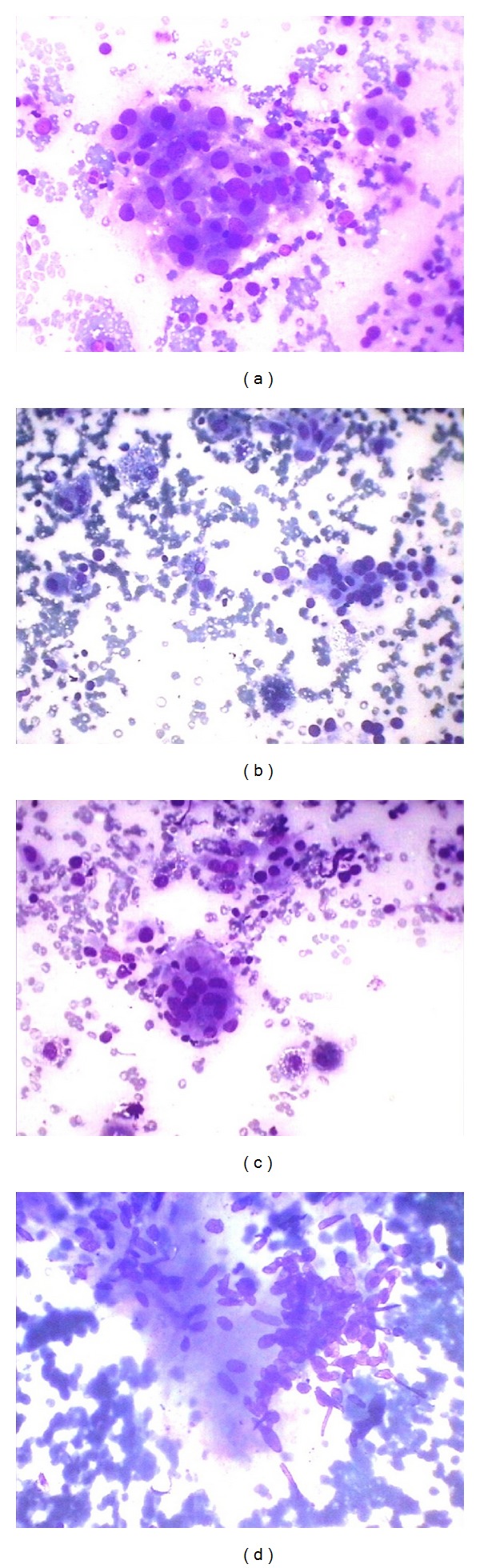
(a) Cluster of follicular epithelial cells showing Hurthle cells changes (Giemsa ×400). (b) Cluster of follicular epithelial cells with pigment laden macrophages (Giemsa ×400). (c) Giant cells with few follicular epithelial cells (Giemsa ×400). (d) Granuloma in lymphocytic thyroiditis (Giemsa ×400).

Out of 175 cases, lymphoid infiltrate was seen in 55 cases. Commonest age group of lymphocytic thyroiditis was 21–30 years with male: female ratio being 1 : 10. These cases were reevaluated in context of grading of lymphoid infiltrate from grade 1 to grade 3 as per the criteria mentioned in Table 1 and were correlated with anti-TPO and hypersensitive TSH. Grade I lymphocytic thyroiditis was observed in twelve (21.82%) cases and showed mild lymphocytic infiltration of follicular epithelial cells, Hurthle cell change, and giant cells. Grade II was observed in seventeen cases (30.91%) and characterized by moderate degree of lymphoid infiltrate with evidence of follicular destruction, Hurthle cell change, giant cells, and so forth. Grade III thyroiditis was noted in twenty-six (47.27%) cases and characterized by dense lymphoid infiltrates with germinal centers and with few residual follicular cells, Hurthle cell change, giant cells, and granulomas. Infiltrate showed polymorphous population of lymphoid cells consisting of mature lymphocytes, centrocytes, centroblast, and plasma cells (Figures 1 and 2). Anti-TPO and TSH were elevated in 96.16% (25/26) of cases with grade 3 lymphoid infiltrate, 94.12% (16/17) of cases with grade 2, and 91.67% (11/12) of cases with grade 1. Increased anti-TPO with raised TSH without any lymphoid infiltrate was seen in 5 cases and 5 cases showed only raised TSH without raised anti-TPO and without any lymphoid infiltrate (Table 2). Statistical correlation of grades of thyroiditis was carried out between them by using SPSS 20 version. We observed that grade 3 lymphocytic infiltration has correlated with TPO and TSH together or TSH alone but not with anti-TPO alone. We also observed that anti-TPO and TSH together are significant even if no lymphocytic infiltration is present. This could be due to very early stage of lymphocytic thyroiditis (Table 3).

## 4. Discussion

Chronic lymphocytic (Hashimoto) thyroiditis is a common autoimmune disease which is characterized by marked lymphoid infiltrate destroying the thyroid follicles with a peak incidence between ages 40 and 60 years and female predominance [4] while Bhatia et al. observed the commonest age group to be the 3rd to 4th decade [2]. In present study, the commonest age group was 21–30 years. Female predominance has been observed in present study similar to other studies [1, 4]. Follicular architecture is totally destroyed and replaced by fibrosis in the long duration of disease [2]. The active phase of disease is transient with clinical manifestations of thyrotoxicosis while the evolution phase and destructive phase manifest with subclinical or overt hypothyroidism [2]. The FNAC smears were characterized by cellular aspirate with numerous dispersed, heterogeneous lymphoid cells and few follicular cells [4]. The diagnosis is established by correlating clinical findings with cytological and serologic test results [4]. The most common circulating autoantibodies are antithyroglobulin and anti-TPO [4]. Antithyroglobulin and anti-thyroid peroxidase levels are significantly higher in the lymphocytic thyroiditis goiter group; however, anti-TPO is increased in greater number of cases in comparison to antithyroglobulin [5, 6]. Anti-TPO autoantibodies can mediate the antibody-dependent cell cytotoxicity on thyrocytes* in vitro* and thyroid destruction is caused by TPO-specific T cells either by direct cytotoxicity mediated by CD4 and CD8 T cells or by programmed apoptosis mediated by Fas and TNF [7]. TPO is present on the apical surface of thyroid follicular cells and is the antigen most closely involved in cell-mediated cytotoxicity and the anti-TPO antibodies titer represents the degree of lymphocytic infiltration of the thyroid gland, reflecting the current activity of HT [8]. TPO is an important enzyme in the synthesis of thyroid hormone and anti-TPO antibodies are helpful in diagnosing and estimating the clinical course of autoimmune thyroid diseases. Measuring circulating antibodies to thyroglobulin to detect autoimmune thyroid disease is uncommon measurement of anti-TPO that gives reliable information about autoimmune thyroid disease [9]. Bhatia et al. conclude that lymphocytic infiltration of thyroid follicles is pathognomic of lymphocytic thyroiditis and emphasised upon it as gold standard but found poor correlation between the grade of thyroiditis and clinical, biochemical, ultrasonographic, and radionuclide parameters [2]. Singh et al. analyze the cytomorphologic spectrum of Hashimoto's thyroiditis on FNAC and correlate cytologic findings with serologic parameters though they observed that grading of thyroiditis and lymphocytic infiltration showed no correlation with the clinical severity of the disease, but a high lymphoid : epithelial ratio was strongly correlated with thyroid peroxidase positivity and thyroid peroxidase positivity is statistically strongly associated with HT as compared to HT coexisting with follicular hyperplasia/Hashitoxicosis/neoplasm [10]. In the present study, the higher the grade of lymphoid infiltrate, the higher the percentage of patients with increased anti-TPO antibodies. The typical patient with hypothyroidism secondary to autoimmune thyroiditis will have an elevated TSH, a low FT4, and positive anti-TPO antibodies; however, in early stages of the disease, TSH may be normal and anti-TPO antibodies may be positive with or without goiter [11]; similarly, in present study, 5 cases showed increased anti-TPO without lymphoid infiltrate and normal TSH level.

## 5. Conclusion

We conclude that the commonest age group of lymphocytic thyroiditis was 21–30 years with male : female ratio being 1 : 10. Grade 3 lymphocytic infiltration has statistical correlation with TPO and TSH together or TSH alone but not with anti-TPO alone. Anti-TPO was adjunct to TSH in grade 3. We also conclude that anti-TPO and TSH together are significant even if no lymphocytic infiltration is present. This could be due to very early stage of lymphocytic thyroiditis. Presence of Hurthle cell change, giant cells, and granulomas has no statistical correlation with anti-TPO and TSH.

## Figures and Tables

**Table 1:** Grading of thyroiditis on cytological material.

Grade	Morphological features	% in our study
Grade 0	No lymphoid cells	15.38
Grade 1	Few lymphoid cells infiltrating the follicles/increased number of lymphocytes in the background	18.47
Grade 2	Moderate lymphocytic infiltration or mild lymphocytic infiltration with Hurthle cell change/giant cells/anisonucleosis	26.15
Grade 3	Florid lymphocytic inflammation with germinal center formation, very few follicular cells left	40

**Table 2:** Grading of lymphocytic thyroiditis and anti-TPO and TSH relation.

	Lymphocyte grade				Total
	0+	1+	2+	3+	
Anti-TPO increase TSH increase	5	3	13	21	42
Anti-TPO increase TSH normal	5	3	2	2	12
Anti-TPO increase TSH low	0	5	0	2	7
Anti-TPO normal, TSH increase	0	1	2	1	4

Total	10	12	17	26	65

**Table 3:** Correlation of grading of lymphocytic thyroiditis with anti-TPO and TSH relation.

	Lymphocyte grade		Chi square with degrees of freedom	*P* value
	1+	0+		
Anti-TPO increase, TSH increase	3	5	0.000, 1	1.000
Anti-TPO increase, TSH normal	3	5		

	2+	0+		
Anti-TPO increase, TSH increase	13	5	2.389, 1	>0.05
Anti-TPO increase, TSH normal	2	5		

	3+	0+		
Anti-TPO increase, TSH increase	21	5	4.858, 1	<0.05
Anti-TPO increase, TSH normal	2	5		
